# Whole-Genome Sequence of “Candidatus Rickettsia asemboensis” Strain NMRCii, Isolated from Fleas of Western Kenya

**DOI:** 10.1128/genomeA.00018-15

**Published:** 2015-03-12

**Authors:** Dereje D. Jima, Alison Luce-Fedrow, Yu Yang, Alice N. Maina, Erik C. Snesrud, Elkanah Otiang, Kariuki Njenga, Richard G. Jarman, Allen L. Richards, Jun Hang

**Affiliations:** a Viral Diseases Branch, Walter Reed Army Institute of Research, Silver Spring, Maryland, USA; b Viral and Rickettsial Diseases Department, Naval Medical Research Center, Silver Spring, Maryland, USA; c Multidrug-Resistant Organism Repository and Surveillance Network (MRSN), Walter Reed Army Institute of Research, Silver Spring, Maryland, USA; d Kenya Medical Research Institute, Nairobi, Kenya

## Abstract

Herein we present the draft genome sequence and annotation of “Candidatus Rickettsia asemboensis” strain NMRCii. “*Ca.* Rickettsia asemboensis” is phylogenetically related to but distinct from the flea-borne spotted fever pathogen Rickettsia felis. “*Ca.* Rickettsia asemboensis” was initially identified in and subsequently isolated from Ctenocephalides cat and dog fleas from Kenya.

## GENOME ANNOUNCEMENT

Rickettsial diseases are endemic worldwide and they can be severe and fatal if diagnosis and antibiotic treatment are delayed (1–3). The causative agents are vectored to humans and animals by various ticks, mites, lice, and fleas. Rickettsia spp. are obligate intracellular Gram-negative bacteria requiring biosafety level 3 procedures and laboratories to work with, which makes it impracticable to use cell culture for routine diagnostics. Serological assays with paired samples and rapid quantitative real-time PCR tests are used for confirmatory diagnosis (4).

Rickettsia felis, a flea-borne spotted fever pathogen, was first identified in the United States and subsequently found in many other countries (5, 6). A number of R. felis and R. felis–like organisms have been detected from a wide range of invertebrate hosts (7) and recently from arthropods in the Asembo division, Siaya County, western Kenya (8). Sequences of *rrs*, *gltA*, *ompA*, *ompB*, *sca4*, and the 17 kDa antigen genes from flea DNA preparations suggested the presence of two rickettsial genotypes, one belonging to R. felis and a new genotype related to R. felis but distinct from it. The differences between this new Rickettsia genotype and other established Rickettsia species satisfied the gene sequence-based criteria to classify it as a new species, designated “Candidatus Rickettsia asemboensis” (8). Prevalence of “*Ca.* Rickettsia asemboensis” in domestic fleas from Asembo was about nine times that of R. felis. Interestingly, all clinical rickettsial infections examined in the area were associated with R. felis and not “*Ca.* Rickettsia asemboensis” (8, 9).

The genome of “*Ca.* Rickettsia asemboensis” strain NMRCii was sequenced by using the MiSeq sequencer (Illumina, San Diego, CA, USA) with the TruSeq DNA PCR-Free shotgun library and paired-end sequencing with the MiSeq Reagent Kit version 3 (600-cycle). A total of 1,976,742 quality-filtered reads, 600 Mb of sequence data, were subjected to *de novo* assembly with the software DeconSeq (10) and the Roche GS *De Novo* assembler (Newbler) version 2.8 followed by contig scaffolding (11). The sequences of rickettsial culture host, Aedes albopictus C6/36 cell line, were detected based on coverage quantitation and BLAST search and removed from the analysis. The draft genome sequence consisted of 88 contigs with sizes ranging from 207 to 86,066 bp and an average sequence alignment coverage of 346-fold. The estimated genome size and G+C content were 1.44 Mb and 32.2%, respectively.

The IGS Annotation Engine was used for whole-genome structural and functional annotation (12). The “*Ca.* Rickettsia asemboensis” genome has 1,147 predicted protein-coding genes, 33 tRNA genes, and 3 *rrn* operons. The features agree with the genome of R. felis (NC_007109), which is 1.49 Mb in size and contains 1,400 protein-coding genes, 33 tRNA genes, and 3 *rrn* operons (7). Of the predicted R. felis proteins, 1,157 (83%) have homologs in “*Ca.* Rickettsia asemboensis.” Further comparative analysis will shed light on the pathogenicity of R. felis and the probability of human infection by “*Ca.* Rickettsia asemboensis.”

### Nucleotide sequence accession numbers.

This whole-genome shotgun project has been deposited at DDBJ/EMBL/GenBank under the accession number JWSW00000000. The version described in this paper is version JWSW01000000.
